# The effect of physical and psychological stress on the oral microbiome

**DOI:** 10.3389/fpsyg.2023.1166168

**Published:** 2023-07-05

**Authors:** Savanna Stoy, Alexandra McMillan, Aaron C. Ericsson, Amanda E. Brooks

**Affiliations:** ^1^Office of Research and Scholarly Activity, Rocky Vista University, Parker, CO, United States; ^2^Department of Veterinary Pathobiology, University of Missouri Metagenomics Center, University of Missouri, Columbia, MO, United States

**Keywords:** oral microbiome, stress response, richness, diversity, dysbiosis

## Abstract

**Background:**

The oral microbiome is incredibly complex, containing a diverse complement of microbiota that has previously been categorized into 6 broad phyla. While techniques such as next-generation sequencing have contributed to a better understanding of the composition of the oral microbiome, the role it plays in human health and disease is still under investigation. Previous studies have identified that a more diverse microbiome is advantageous for health. Therefore, alterations to the physical or mental health that are of interest in this study, such as stress, are the factors that decrease microbial diversity, leading to the potential for dysbiosis and disease disposition. Intensive Surgical Skills Week (ISSW) is a hyper-realistic simulation training week for military medical students that takes place at the Strategic Operations (STOPS) facility in San Diego, CA. This training week puts students through mass causality simulations and requires them to work through distinct roles within the healthcare team, providing an almost ideal environment to assess the impact of acute stress on oral microbiome diversity. Based on the literature on stress and microbiota, we hypothesized that the high stress simulation events at ISSW will impact the composition and diversity of the oral microbiome.

**Methods:**

To investigate this hypothesis, thirty-seven (*n* = 37) second-or third-year medical students who are enlisted in a branch of the military and who attended ISSW in July of 2021 were included in the study. Student participants were divided into 7 teams to complete the hyper-realistic simulations (SIMs) at ISSW. A pilot of sixty-four buccal samples (*n* = 64) from three of the seven teams were sent for analysis at the University of Missouri Metagenomic Center.

**Results:**

We saw an overall increase in species richness at the end of ISSW when looking at all samples (*n* = 64). Fourteen significantly different bacteria were identified from the beginning to the end of data collection. Additionally, third year medical students appear to have a greater species richness compared to second year medical students. Further, third year medical students had a statically significant difference in their oral microbiome richness from beginning to end of data collection (*p* = 0.008).

**Conclusion:**

Our preliminary data indicates that physical and psychological stress can impact the composition of the oral microbiome. The analyses in this study show that using the oral microbiome as an indicator of stress is promising and may provide evidence to support stress management practices.

## Introduction

All the microorganisms that make up a specific environment are known as microbiomes and there are several that exist on or within the human body including: skin, respiratory tract, genitourinary tract, and the gastrointestinal tract. Each microbiome found on the human body demonstrates variable composition by anatomical location.

This study aims to explore the oral microbiome as a component of the gastrointestinal tract, and specifically looks at the microbiome of the buccal mucosa. The oral microbiome is incredibly complex containing a diverse environment of microbiota that has previously been categorized into 6 broad phyla including Firmicutes, Actinobacteria, Proteobacteria, Fusobacteria, Bacteroidetes, and Spirochetes (Wade, 2013; Verma et al., 2018). These 6 phyla make up 96% of the core microorganisms found in the oral cavity and are considered healthy or commensal (Verma et al., 2018).

The composition of microorganisms within the oral cavity has previously been described to have a direct influence on human health. Microbiota dysbiosis, or a state of unbalance, have been associated with periodontal disease and other systemic diseases such as Alzheimer’s disease, diabetes, and cancer (Duran-Pinedo et al., 2018; Verma et al., 2018; Sureda et al., 2020). The concept that oral bacteria may serve as biomarkers for non-oral diseases highlights the implications the composition of the oral microbiome has beyond oral health (Krishnan et al., 2017). This makes the oral microbiome of clinical interest and highlights its potential in the future of health management.

## Oral microbiome and stress

When we are considering what makes a healthy microbiome, diversity is at the forefront. Previous studies have identified that a more diverse microbiome is advantageous for health. Therefore, alterations that are of interest in this paper are the factors that decrease microbial diversity leading to the potential for dysbiosis and disease disposition. Many factors can alter the composition of the microbiome including different disease states and lifestyle elements including diet and smoking (Wade, 2013; Verma et al., 2018). Additionally, host-stress, both acute and chronic, have been shown to alter the oral microbiome (Gur et al., 2015). Our focus will be on the affect both physical and psychological stress has on the diversity of the oral microbiome.

The effect of stress has been examined in a previous study which found cortisol, a stress hormone secreted in saliva, altered the profile of microbiota within a dental plaque to resemble that of periodontitis progression (Duran-Pinedo et al., 2018). We would like to expand the knowledge of the role stress plays in microbial composition outside the limited scope of dental disease. Additionally, recent studies have analyzed the effects of exercise-induced stress on the gut-brain axis, a bidirectional link between the central nervous system and the enteric nervous system (Bienenstock et al., 2015). There is a high correlation between exercise induced stress and changes in the gastrointestinal microbiome; however, the effects of exercise induced stress on the oral microbiome remains understudied (Clark and Mach, 2016).

The diversity of the oral microbiome highlights the importance of understanding the associations between composition and disease risk. Microbiome impacts metabolism and immune response (Verma et al., 2018). Little research has been done in this field, making our research question novel and relevant. Assessing induced stress and its effect on the oral microbiome will increase understanding of the shift in microbiota that takes place *in vivo* and the implications of these changes.

## Intensive Surgical Skills Week

Intensive Surgical Skills Week (ISSW) is a hyper-realistic simulation training week for military medical students that takes place at the Strategic Operations (STOPS) facility in San Diego, CA. This training week puts students through mass causality simulations and requires them to work through distinct roles within the healthcare team. In our study, we focus on the emergency department and the operating room as they are considered the most stressful SIM events. The roles each student can play include triage, emergency department physician, patient tracking, surgical resident, scrub nurse, 1st assist, medical students, and anesthesiologist. The unique aspect of this training is the incorporation of Surgical Cut Suits, which are realistic human suits that can mimic severe traumatic events while being worn by an actor.

In addition to simulation exercises, students are required to master several medical techniques and give a grand rounds presentation over a pre-determined medical topic. ISSW relies on stress inoculation to build resilience and provides a microcosm of extremely stressful situations in a controlled setting in which to conduct research. The nature of this training provides a standardized way to reproduce realistic events in a way that does not endanger participants or others while inducing extreme stress.

Additionally, previous studies at ISSW have demonstrated the hyper-realistic simulations experienced during the week altered cortisol levels. Since cortisol has been shown to alter microbial composition, we are interested in directly exploring the impact of stress on the oral microbiota outside the realm of dental plaques or dental disease (Duran-Pinedo et al., 2018). Our hypothesis concluded that the high stress simulation events at ISSW will impact the composition of the oral microbiome.

## Materials and methods

### Subjects

The study included 37 subjects (*n* = 37) that were all second- or third-year medical students currently enlisted in a branch of the military and enrolled at different medical schools across the United States. Subjects were randomly divided into seven teams, except for team 1 which was selected to only have third year medical students. Each student was given a research ID at the beginning of the week so all data would remain anonymous.

### Experimental design

**FIGURE 1:**
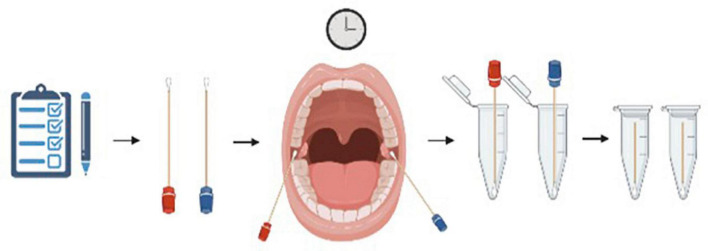
Visual representation of the collection process each subject went through. A buccal swab was collected from both the left and right buccal mucosa simultaneously. Each subject was given both a wet (blue) and a dry (red) swab for each timepoint and instructed to vigorously rub their buccal surface near their molars for 30 s. Swabs were then placed into pre-labeled microcentrifuge tubes and placed in a freezer at –20°C. Figure created using BioRender.

At seven different time points, each participant completed a subjective questionnaire (Appendix 1) and a buccal swab was collected from both the left and right buccal mucosa simultaneously. Timepoints included baseline (T0) and then immediately after the most stressful SIM event of the day (T1, T2, T3, T4, and T5), and an end baseline (T6). Sterile cotton swabs were used for the collection of buccal samples. Each subject was given both a wet and a dry swab for each timepoint. Wet and dry swabs were handed randomly to either the left or right hand of each subject to not favor one side of the buccal mucosa with either the wet or the dry swab. Each wet swab was rinsed five times in filtered water to ensure the cotton was saturated with water. Subjects were instructed to vigorously rub their buccal surface near their molars for 30 s. Swabs were then placed into pre-labeled microcentrifuge tubes and placed in a freezer at –20°C (Figure 1).

A total of 64 buccal samples were collected for analysis from three of the seven teams. The three teams were chosen based on demographics and an ISSW schedule that would allow for the greatest number of analyses. Team 1 consisted of five third year medical students, team 3 consisted of 6 s year medical students and team 7 consisted of 5 s year medical students (Appendix 2). We chose team 1 because they were the only team with all third-year medical students. We chose team 3 because their group had both stressful simulation events on the same day. Lastly, we chose team 7 as well as team 1 because their groups had the stressful simulation events on separate days. For each subject and timepoint, only the wet or the dry sample was analyzed. This decision was made based on the reported mouthfeel of the subject at the time of sample collection as indicated by the subjective questionnaire. If the subject selected mouthfeel in the 1–3 range, indicating there was no excessive dryness of the oral cavity, the dry sample was selected. If the subject chose 4 or 5, indicating dryness of the oral cavity, the wet sample was selected. All samples (*n* = 64) were sent to the University of Missouri Metagenomic Center for DNA extraction and preparation.

### DNA extraction

Deoxyribonucleic acid was extracted using PowerFecal kits (Qiagen) according to the manufacturer instructions, with the exception that samples were homogenized in the provided bead tubes using a TissueLyser II (Qiagen, Venlo, Netherlands) for 10 min at 30/sec, rather than performing the initial homogenization of samples using the vortex adapter described in the protocol, before proceeding according to the protocol and eluting in 100 μL of elution buffer (Qiagen). DNA yields were quantified via fluorometry (Qubit 2.0, Invitrogen, Carlsbad, CA, USA) using quant-iT BR dsDNA reagent kits (Invitrogen) and normalized to a uniform concentration and volume.

## 16S rRNA library preparation and sequencing

Library preparation and sequencing were performed at the University of Missouri (MU) Genomics Technology Core. Bacterial 16S rRNA amplicons were constructed via amplification of the V4 region of the 16S rRNA gene with universal primers (U515F/806R) previously developed against the V4 region, flanked by Illumina standard adapter sequences (Caporaso et al., 2011; Walters et al., 2011). Dual-indexed forward and reverse primers were used in all reactions. PCR was performed in 50 μL reactions containing 100 ng metagenomic DNA, primers (0.2 μM each), dNTPs (200 μM each), and Phusion high-fidelity DNA polymerase (1U, Thermo Fisher). Amplification parameters were 98°C (3 min) + [98°C (15 s) + 50°C (30 s) + 72°C (30 s)] × 25 cycles + 72°C (7 min). Amplicon pools (5 μL/reaction) were combined, thoroughly mixed, and then purified by addition of Axygen Axyprep MagPCR clean-up beads to an equal volume of 50 μL of amplicons and incubated for 15 min at room temperature. Products were then washed multiple times with 80% ethanol and the dried pellet was resuspended in 32.5 μL EB buffer (Qiagen), incubated for 2 min at room temperature, and then placed on the magnetic stand for 5 min. The final amplicon pool was evaluated using the Advanced Analytical Fragment Analyzer automated electrophoresis system, quantified using quant-iT HS dsDNA reagent kits, and diluted according to Illumina standard protocol for sequencing as 2 × 250 bp paired-end reads on the MiSeq instrument.

## Informatics analysis

Deoxyribonucleic acid sequences were assembled and annotated at the MU Informatics Research Core Facility. Primers were designed to match the 5’ ends of the forward and reverse reads. Cutadapt (version 2.6) was used to remove the primer from the 5’ end of the forward read (Martin, 2011). If found, the reverse complement of the primer to the reverse read was then removed from the forward read as were all bases downstream. Thus, a forward read could be trimmed at both ends if the insert was shorter than the amplicon length. The same approach was used on the reverse read, but with the primers in the opposite roles. Read pairs were rejected if one read or the other did not match a 5’ primer, and an error-rate of 0.1 was allowed. Two passes were made over each read to ensure removal of the second primer. A minimal overlap of three bp with the 3’ end of the primer sequence was required for removal.

The QIIME2 DADA2 plugin (version 1.10.0) was used to denoise, de-replicate, and count ASVs (amplicon sequence variants), incorporating the following parameters: (1) forward and reverse reads were truncated to 150 bases, (2) forward and reverse reads with number of expected errors higher than 2.0 were discarded, and (3) Chimeras were detected using the “consensus” method and removed (Callahan et al., 2016; Bolyen et al., 2019). R version 3.5.1 and Biom version 2.1.7 were used in QIIME2. Taxonomies were assigned to final sequences using the Silva.v132 database, using the classify-sklearn procedure (Pruesse et al., 2007; Kaehler et al., 2019).

## Results

**FIGURE 2:**
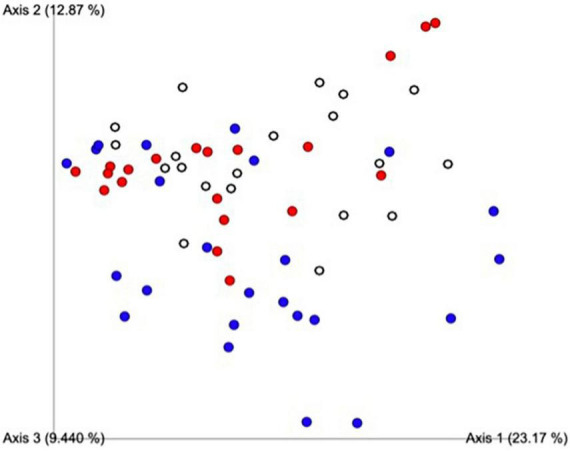
Principal component analysis of all data samples (*n* = 64). Colors indicate samples by team. Red includes all samples from Team 1, blue from Team 3, white from Team 7. This graph was formed using the Qiime2veiw program and a Bray-Curtis Emperor plot. Each axis refers to the percentage of variation explained by each axis of the ordination.

**FIGURE 3:**
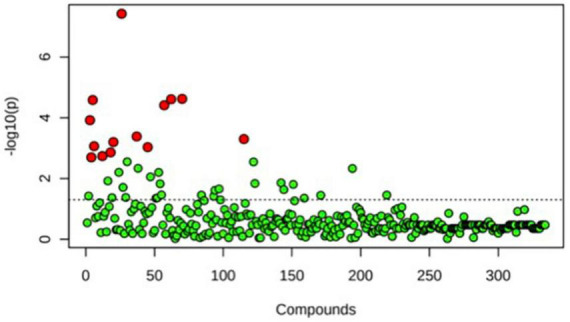
One-way ANOVA comparing samples from each participant. The ANOVA analysis identified 14 significantly different samples between all participants (*n* = 14) and are shown in red.

**FIGURE 4:**
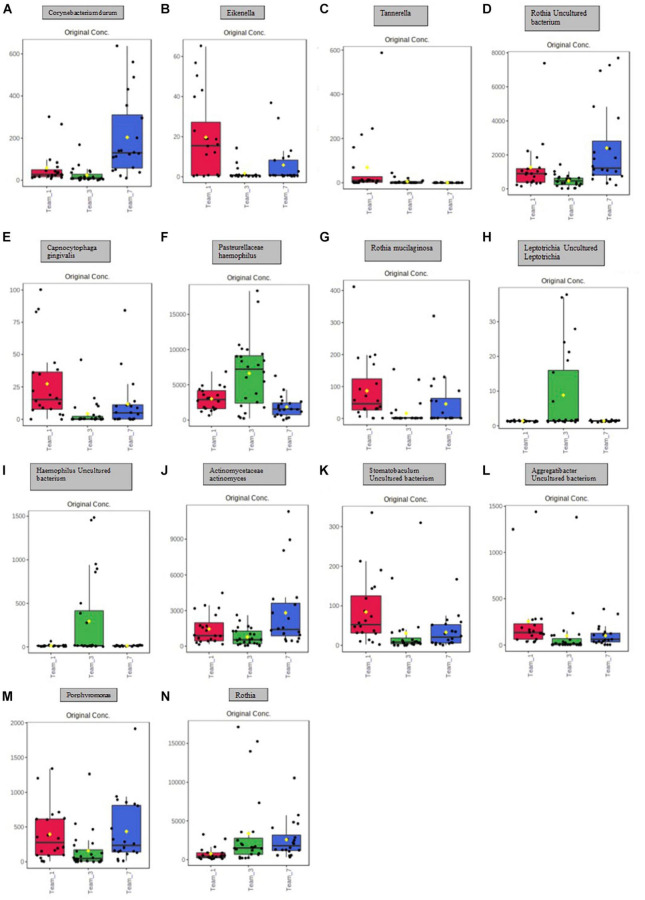
Boxplot representation of the 14 significant points identified in Figure 5 and Table 1. Significance is denoted by the yellow circle. **(A)** Significant difference between Team 1 and Team 3; Team 1 and Team 7; Team 3 and Team 7. **(B)** Significant difference between Team 1 and Team 3; Team 3 and Team 7. **(C)** Significant difference between Team 1 and Team 3; Team 3 and Team 7. **(D)** Significant difference between Team 1 and Team 3; Team 1 and Team 7; Team 3 and Team 7. **(E)** Significant difference between Team 1 and Team 3; Team 1 and Team 7; Team 3 and Team 7. **(F)** Significant difference between Team 1 and Team 3; Team 3 and Team 7. **(G)** Significant difference between Team 1 and Team 3; Team 3 and Team 7. **(H)** Significant difference between Team 1 and Team 3; Team 3 and Team 7. **(I)** Significant difference between Team 1 and Team 3; Team 3 and Team 7. **(J)** Significant difference between Team 3 and Team 7. **(K)** Significant difference between Team 1 and Team 3; Team 1 and Team 7. **(L)** Significant difference between Team 1 and Team 3; Team 1 and Team 7. **(M)** Significant difference between Team 1 and Team 3; Team 3 and Team 7. **(N)** Significant difference between Team 1 and Team 3; Team 1 and Team 7.

**FIGURE 5:**
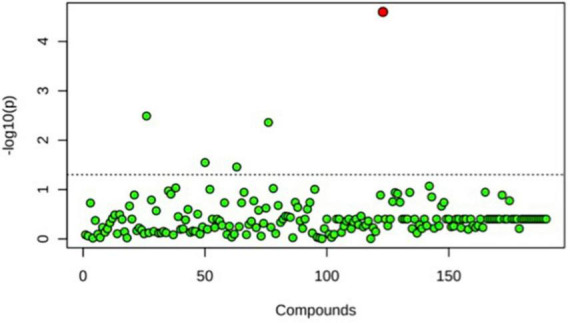
One-way ANOVA comparing T0, T4, and T6 for Team 1. Only one significant difference was detected. This was seen at T6, the end point baseline. No other significant differences between time points were detected.

**FIGURE 6:**
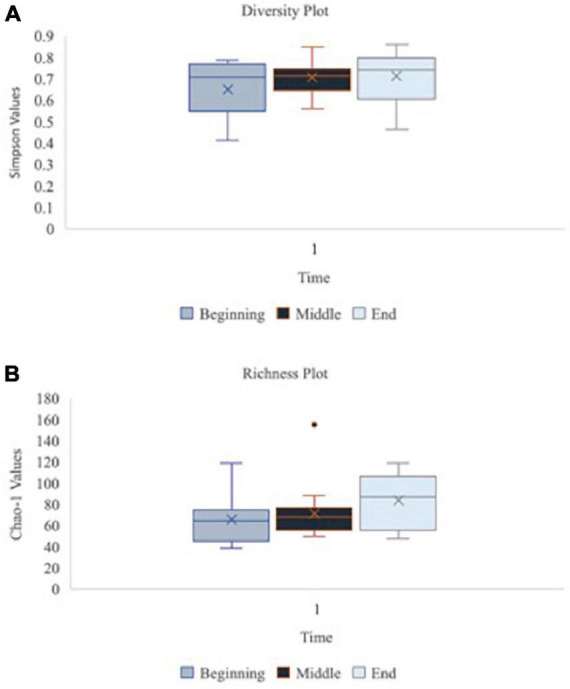
Oral microbiome diversity and richness plots at the baseline, middle and endpoint of data collection for teams 1, 3, and 7 (*n* = 16 at each time point). Graph **(A)** illustrates the diversity of species in the oral microbiome using Simpson function at the beginning, middle vs. end of data collection for teams 1, 3, and 7 (*n* = 16 at each time point). Simpson diversity index measures the diversity of species in a community. Graph **(B)** depicts the richness of species in the oral microbiome using Chao-1 values at beginning, middle vs. end of data collection for teams 1, 3, and 7 (*n* = 16 at each time point). Chao-1 represents an estimate of species richness based on abundance.

**FIGURE 7:**
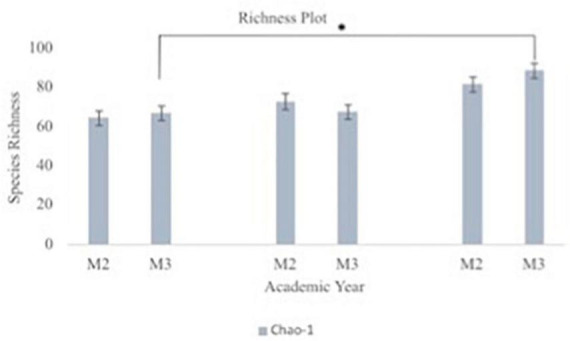
Oral microbiome species richness plots between second year (M2) and third year (M3) medical students. The graph depicts Chao-1 species richness from baseline to each team’s midpoint of data collection to the end of data collection for second year vs. third year medical students. Chao-1 represents an estimate of species richness based on abundance. Third year medical students had statistically significant difference in their oral microbiome richness from beginning to end of data collection (*p* = 0.008).

A principal component analysis of all data samples (*n* = 64) was formed using Qiime2veiw program and a Bray-Curtis Emperor plot to illustrate the percentage of variation between the samples (Figure 2). From the samples (*n* = 64), 14 significantly different bacteria were identified and are shown in red (Figure 3). These include species from several different genera including *Corynebacterium*, *Eikenella*, *Tannerella*, *Rothia*, *Capnocytophaga*, *Pasteurellaceae*, *Leptotrichia*, *Haemophilus*, *Actinomycetaceae*, *Stomatobaculum*, *Aggregatibacter*, and *Porphyromonas*. A list of these genera is shown along with their value of significance in Table 1. Since the significant *p*-values were so small, an inverse Log_10_ was used to bring the numbers into a usable and comprehensible form, allowing for better interpretation and visualization. Some species are not included following their genus due to the uncultured nature of these specific species of the bacterial genus. The boxplots represent the significant data points and include the teams in which a significant difference was observed (Figure 4). Team 1 was the only team that showed a significant difference among their group over the collection timepoints; the clinical significance of that point is unknown (Figure 5). One outlier point at a position toward the right of the graph implies little true significance for the solitary bacterial group (Table 2). For teams that did not show a significant difference in their oral microbiome composition, ANOVA plots can be referenced in the supplemental data. Diversity and richness were plotted for teams 1, 3, and 7 at the beginning, middle and end of data collection as seen in Figure 6. Simpson diversity index measures the diversity of species in a community. The higher the value of Simpson diversity index, the higher the diversity. In the graph measuring the Simpson diversity index, species diversity was increased at the end of data collection as opposed to the first and middle samples collected for each team. Additionally, richness of the species was compared from beginning, middle and end of data collection for teams 1, 3, and 7. Chao-1 represents an estimate of species richness based on abundance. Species richness increased at the end of data collection in comparison to the first and middle samples collected for each team. We then compared the richness between the 2nd year vs. the 3rd year medical students at the beginning, middle and end of data collection as depicted in Figure 7. At the beginning of buccal sample collection (*T* = 0), the 3rd year students had greater species richness compared to the 2nd year students. Whereas, at the mid-point of data collection (*T* = 4, *T* = 3, *T* = 2) for teams 1, 3, 7, the 2nd year students had an increased species richness compared to the 3rd year students. However, at the end of data collection (*T* = 5), the 3rd year students’ buccal samples had more richness than the 2nd year students’ samples.

**TABLE 1:** Important features identified by One-way ANOVA and *post hoc* analysis between Team 1, Team 3, and Team 7.

Boxplot	Compound	*P*-value	-log_10_ (p)	Genus	Species
A	ASV0026	0.00	7.43	Corynebacterium	Durum
B	ASV0070	0.00	4.62	Eikenella	
C	ASV0062	0.00	4.61	Tannerella	
D	ASV0005	0.00	4.59	Rothia	Uncultured bacterium
E	ASV0057	0.00	4.41	Capnocytophaga	Gingivalis
F	ASV0003	0.00	3.92	Pasteurellaceae	Haemophilus
G	ASV0037	0.00	3.39	Rothia	Mucilaginosa
H	ASV0115	0.00	3.3	Leptotrichia	Uncultured Leptotrichia*
I	ASV0020	0.00	3.2	Haemophilus	Uncultured bacterium
J	ASV0006	0.00	3.06	Actinomycetaceae	Actinomyces
K	ASV0045	0.00	3.03	Stomatobaculum	Uncultured bacterium
L	ASV0018	0.00	2.86	Aggregatibacter	Uncultured bacterium
M	ASV0012	0.00	2.74	Porphyromonas	
N	ASV0004	0.00	2.7	Rothia	

*Represents that some species are not included following their genus due to the uncultured nature of these specific species of the bacterial genus.

**TABLE 2:** Important features identified by One-way ANOVA and *post hoc* analysis between beginning, middle, and end timepoints for team 1.

Number	Compounds	*P*-value	-log_10_ (p)	Genus	Species
1	ASV0130	0.00	4.6	(Eubacterium) yurii group	Uncultured bacterium

The clinical significance of this point is uncertain.

## Discussion

We saw an overall increase in species richness at the end of ISSW when looking at all samples (*n* = 64). Additionally, third year medical students appear to have greater richness in their oral microbiomes when compared to second year medical students. The 14 significantly different bacteria identified have various pathogenic potential and can be isolated from various areas of the body. The species *Corynebacterium durum* has been cultured from throat, respiratory secretions, and blood (Bernard, 2012). The pathogenic potential of *Corynebacterium* durum remains unclear; however, recent studies on polymicrobial interactions with oral mucosal surfaces have determined *C. durum* functions as a commensal species (Redanz et al., 2021). The genus *Eikenella* is a pathogen in head and neck infections and in wounds related to oral cavity contamination (Knudsen and Simko, 1995). *Eikenella* can cause persistent peritonsillar abscesses and is resistant to clindamycin; therefore, the treatment of choice is antibiotic therapy with penicillin (Knudsen and Simko, 1995). The genus *Tannerella* is associated with subgingival plaque development and with progressive periodontitis (Potempa, 2013). The genus *Rothia* are opportunistic pathogens associated with various infections in immunocompromised and immunocompetent individuals, including bacteremia, endocarditis, meningitis, peritonitis, bone and joint infections, pneumonia, skin, and soft tissue infection, endophthalmitis, and prosthetic device infections (Ramanan et al., 2014; Fatahi-Bafghi, 2021). The species *Rothia dentocariosa* and *Rothia mucilaginosa* are colonizers of the oral microbiome and have been isolated from dental plaques in periodontal disease (Rose and Reboli, 2015). The genus *Capnocytophaga* have been reported as causative agents of periodontitis, dental abscess and necrotizing ulcerative gingivitis (Newman, 2019). The species *Capnocytophaga gingivalis* has rarely been reported as a cause of disease in immunocompromised or immunocompetent hosts; however, in a recent case study, a 70-year-old immunocompromised man suffered *C. gingivalis* bacteremia after an episode of upper gastrointestinal bleeding (Lawal and Baer, 2021). The pathogenic species of the family *Pasteurellaceae* can be cultured from mucosal membranes of the oral cavity, respiratory, and genital tracts (Knoop, 2007). The genus *Pasteurellaceae haemophilus* is most associated with systemic disease including bacteremia, pneumonia, epiglottis, and acute bacterial meningitis (Knoop, 2007). The genus *Leptotrichia* colonizes the oral cavity and genital tract and is most commonly commensal organisms; however, in immunocompromised individuals with neutropenia the genus can become pathogenic and a rare cause of bacteremia (Eribe and Olsen, 2008). The genus *Haemophilus* cause a variety of clinical syndromes including pneumonia, bacterial endocarditis, chancroid, meningitis, cellulitis, and epiglottitis (Musher, 1996). The most common oral *Haemophilus* species cultured in dental plaques are *Haemophilus parainfluenzae*, *Haemophilus segnis*, and *Haemophilus aphrophilus* (Liljemark et al., 1984). The species *Actinomycetaceae actinomyces* is known to have low virulence and is a normal inhabitant of the mouth and gastrointestinal tract. Most infections with Actinomyces are polymicrobial and can cause plaque formation and periodontal disease (Murray, 2016). Actinomycosis, a pulmonary disease, can occur most commonly from *Actinomyces israelii* from aspiration of oral secretions (Bush and Vazquez-Pertejo, 2022). The genus *Stomatobaculum* has one known species *Stomatobaculum longum* which has been isolated from dental plaques, further research on the pathogenicity of the species is unknown (Sizova et al., 2013). *Aggregatibacter* species are normal inhabitants of the oropharyngeal flora and in dental plaques and are now considered a dominant etiology of infective endocarditis as a member of HACEK organisms, specifically *Aggregatibacter actinomycetemcomitans* (Nørskov-Lauritsen, 2014). *Aggregatibacter actinomycetemcomitans* can be cultured from one-third of healthy adults; however, is the most common organisms detected in periodontitis (Nørskov-Lauritsen et al., 2019). The genus *Porphyromonas*, specifically the species *Porphyromonas gingivalis*, is also found in the oral cavity and associated with periodontal disease as well as systemic, vascular, and inflammatory diseases (Walkenhorst et al., 2020). Despite these strong preliminary data several weaknesses in the collection and the uniqueness of the COVID-19 global environment during their collection compromise the data’s ability to be extended and several outstanding questions remain to be elucidated with this proposal. One of the weaknesses in the study could potentially be contributed to xylitol in chewing gum. For a simultaneous research study during Cut Suit Week, the same participants chewed gum containing xylitol, which has been shown to reduce the levels of mutans streptococci and lactobacilli in saliva and plaque (Rafeek et al., 2018). Additionally, based on the data collected from the 14 significant bacteria identified, it will be important to gather information on previous comorbidities involving vascular diseases and head and neck infections from participants in future studies. Lastly by having the participants complete the subjective questionnaire immediately following each simulation event, we hoped to limit the effect of recall bias that might occur if subjects were asked about their experience and stress level with an increased lapse in time from the event to the questionnaire. Regardless, it is important to note that the questionnaire is subjective and can be influenced by biases held by each participant, a factor that our study did not aim to control. This can be seen as a limitation in this study, as social desirability for medical preparedness could influence answers. Since measures from the questionnaire were not the primary point of the investigation, we see the results of oral microbiome composition as valid and reliable independent of the perceived stress of each individual. Although progress has been made in studying the complex bacteria that inhabits the oral microbiome, the contributions from fungi, viruses, and candidate phyla radiation group (CRP) of ultrasmall bacteria warrants further research (Sureda et al., 2020).

## Conclusion

The analyses in our pilot study show that using the oral microbiome as an indicator of stress is promising and may provide evidence to support stress management practices for groups at a higher risk of associated negative health outcomes of stress. This is still a new and advancing area of research and more evidence is needed to fully understand the impact physical and psychological stress has on the diversity and richness of the oral microbiome, and the role that altered diversity and richness can play is disease predisposition. This study will be repeated following the described protocol to further identify associations between the oral microbiome and physical and psychological stress.

## Data availability statement

The original contributions presented in this study are publicly available. This data can be found here: https://www.ncbi.nlm.nih.gov/bioproject/, PRJNA936174.

## Ethics statement

The studies involving human participants were reviewed and approved by the Rocky Vista University Institutional Review Board. The patients/participants provided their written informed consent to participate in this study.

## Author contributions

All authors listed have made a substantial, direct, and intellectual contribution to the work, and approved it for publication.

## Appendix

### Appendix 1: Participant survey

Name: ____________________________________ Code:________________

Age:_______

Gender:________________________

Year (circle one): MS-II or MS-III

Group: ____________________________________

Role Played in Scenario: ______________________

Do you use tobacco products (circle one): Yes or No.

Have you used any allergy medications in the past 24 h (circle one): Yes or No.

Have you taken antibiotics in the past 24 h (circle one): Yes or No.

Have you used antiseptic mouthwash in the past 24 h (circle one): Yes or No.

Have you eaten anything in the past 30 min (circle one): Yes or No.

Have you drank anything in the past 30 min (circle one): Yes or No.

Do you have previous experience as a first responder (circle one): Yes or No.

If yes, what: __________________________________________________________

Do you have previous military experience (circle one): Yes or No.

On a scale of 1–5, how dry is your mouth currently (circle one):

**Table:**

1	2	3	4	5
No dryness	Mild dryness	Moderate dryness	Much dryness	Extreme dryness-Cotton mouth

On a scale of 1-5, how stressed do you currently feel (circle one):

**Table:**

1	2	3	4	5
No stress	Mild stress	Moderate stress	Much stress	Extreme stress

### Appendix 2: Team demographic information

**Table:**

	Team 1		Team 3		Team 7		Total	
	*n*	%	*n*	%	*n*	%	*n*	%
Gender								
Female	3	60	1	16.7	3	60	7	43.75
Male	2	40	5	83.3	2	40	9	56.25
Education level								
M2	0	0	6	100	5	100	11	68.75
M3	5	100	0	0	0	0	5	31.25
Prior military experience								
Yes	0	0	0	0	0	0	0	0
No	5	100	6	100	5	100	16	100

There were 16 total participants (*n* = 16) in the three teams from which buccal samples were collected at the defined timepoints. Subjects were randomly assigned to each team, apart from Team 1, which contained all third-year medical students (M3).
